# Nursing work environment, professional self‐actualization and marketing of the nursing profession: Cross‐sectional study

**DOI:** 10.1002/nop2.644

**Published:** 2020-10-03

**Authors:** Yelena Hazanov, Yulia Gehman, Rachel Wilf Miron, Ilya Kagan

**Affiliations:** ^1^ Pat Matthews Academic School of Nursing Hillel Yaffe Medical Center Hadera Israel; ^2^ Intensive Cardiac Care Unit Hillel Yaffe Medical Center Hadera Israel; ^3^ Department of Health Promotion School of Public Health Sackler Faculty of Medicine Tel Aviv University Tel Aviv Israel; ^4^ The Gertner Institute for Epidemiology and Health Policy Research Ramat Gan Israel; ^5^ Nursing Department Steyer School of Health Professions Sackler School of Medicine Tel Aviv University Tel Aviv Israel

**Keywords:** marketing of nursing, nurses, nursing work environment, professional self‐actualization, promotion of nursing

## Abstract

**Aim:**

To explore associations between nurses' sense of professional self‐actualization, nursing work environment and their involvement in promotion and marketing of the nursing profession.

**Design:**

A descriptive cross‐sectional study.

**Methods:**

144 nurses from various clinical fields completed a promotion and marketing activity questionnaire, selected items from the Brief Index of Self‐Actualization and a part of the Revised Nursing Work Index.

**Results:**

Nurses' perception of their work environment and holding a master's degree in nursing were associated with their involvement in promotion activities [*R* ^2^ = 0.27, *F*(4, 113) = 10.4, *p* < .001]. Nurses' perception of their work environment was associated with their sense of professional self‐actualization [*R* ^2^ = 0.21, *F*(2, 121) = 16.5, *p* < .001]. Nurses' sense of professional self‐actualization did not contribute to the explanation of variance in their involvement in promotion of the nursing profession.

## 1 INTRODUCTION

The nursing profession is experiencing a growing shortage of workforce worldwide. It has been suggested that an appropriate public image building of the nursing profession may provide solution to the nursing shortage (Drennan & Ross, 2019; Somers et al., 2010). Recently, nursing literature has called for promoting of the nursing profession with the aim to improve the public image of nurses in a variety of ways, especially in the media (Avila et al., 2013; Drennan & Ross, 2019; Hoeve et al., 2014; Price et al., 2019). The task is challenging. Nurses are underrepresented in media and little identified as expert sources by journalists and journals. Two significant studies with similar design and objectives—The Woodhull study by STTI (1997) and 20‐year‐later follow‐up replicative study by Mason et al. (2018)—reported that nurses served as an expert source of information in only 4% and 2% of newspaper health news stories, respectively.

According to the American Marketing Association (2013), “marketing is the activity, set of institutions and processes for creating, communicating, delivering and exchanging offerings that have value for customers, clients, partners and society at large.” The purpose of marketing is to influence the public perception of an organization and its services and to attract and retain customers (Sharma et al., 2010). In the context of nursing, marketing entails nurses' communication to the public of their professionalism and unique contribution to the quality of care and to the global and national public health. Marketing and promotion of the nursing profession may be targeted at the public, but also at policy makers and stockholders, other healthcare professionals and nurses themselves (Kagan et al., 2015). Marketing has been shown to have a positive impact on the public image of nursing as a profession. Thus, in the United States, national efforts to portray more accurately the importance of nurses through mass media have transformed nursing into an attractive career choice (Aiken & McHugh, 2014).

Less is known to what extent individual nurses are involved in promotion and marketing of the nursing profession. Moreover, little is known about the antecedents of nurses' involvement in promotion and marketing activities. The previous study revealed that nurses refrain from active involvement in activities to market the nursing profession to the public or to other target audiences. However, nurses who were more satisfied at work were also more involved in the promotion and marketing activity. Moreover, it was hypothesized that an association might exist between work environment and organizational climate and nurses' involvement in the promotion and marketing of the profession (Kagan et al., 2015). However, this hypothesis requires confirmation.

Nurses' professional self‐actualization has been linked to important concepts such as nurses' work motivation (Gaki et al., 2013; Peters et al., 2010; Toode et al., 2014), job satisfaction and job retention (Banks & Bailey, 2010; Gilles et al., 2014; McGlynn et al., 2012). These associations suggest that nurses' professional self‐actualization may be related to their involvement in promotion and marketing of the nursing profession. The concept of professional self‐actualization is rarely mentioned in the nursing literature and lacks a clear definition. Maslow (1995) defines self‐actualization as the highest order human need, described as an individual's expression of his full potential and a desire for self‐fulfilment and is considered as part of the self‐actualization process in a certain professional field. Professional self‐actualization is an individual's need to develop his/her potential; it is learning and growing in the work environment (Maslow, 2000). Perfilyeva (2012) suggested several indicators of professional self‐actualization. Some of these are an individual's feeling of being professionally competent, perceived importance of the professional activity, experiencing professional self‐esteem, striving for professional growth and personal development, carrying responsibility for the result and work process, being able to make decisions independently and experiencing work satisfaction.

Self‐actualization phenomenon and its characteristics are also discussed in nursing literature. For example, according to Paris and Terhaar (2010), characteristics of the highest level of nursing practice environment needs, corresponding to self‐actualization as described by Maslow, are autonomy, empowerment, decision‐making ability and control over practice (Paris & Terhaar, 2010). Similarly, according to McGlynn et al. (2012), some characteristics of professional nursing practice, enabling nurse to achieve professional self‐actualization, are achievement, advancement, recognition, responsibility and growth (McGlynn et al., 2012). Others relate to feelings of competence and achievement at work (Emold et al., 2011). Associations between professional self‐actualization and job satisfaction (Banks & Bailey, 2010; Burtson & Stichler, 2010; Gilles et al., 2014) and life meaningfulness at work (Burtson & Stichler, 2010) were reported.

The antecedents of nurses' experiencing self‐actualization are even less clear. Literature suggests that nurses' work environment may play an important role in nurses' attaining professional self‐actualization. The well‐known Maslow's pyramid of human needs places the need to self‐actualization at the highest level—the apex as a supreme need. Once a need of a lower level is fulfilled, the need of the next level occupies an individual's attention, until it is satisfied. According to this model, the motivation to meet advanced needs depends on fulfilment of more basic needs of the lower levels. In the context of nurses, to achieve self‐realization at work, it is of great significance to fulfil the lower needs related to positive and supportive work environment (Paris & Terhaar, 2010). Here, literature assigns an important role to the nursing managers, who can foster professional self‐actualization by creating suitable working conditions and thus satisfy nurses' lower order professional needs (Paris & Terhaar, 2010), or by directly fostering staff nurses' professional self‐actualization. Casida and Parker (2011) claimed that by using mentoring, advising, coaching and supporting, nursing managers could mobilize or persuade staff nurses to achieve more and strive towards excellence.

The empirical evidence of the role of nurses' work environment in the development of their sense of professional self‐actualization is limited. However, there is an evidence of association between certain characteristics of the work environment and nurses' feeling of personal accomplishment (Emold et al., 2011; Khamisa et al., 2015; Papathanassoglou et al., 2012), which is a somewhat related term to professional self‐actualization.

Consequently, the literature review raised several research questions: What is the role of nursing work environment in nurses' involvement in promotion and marketing of the nursing profession? What factors are associated with nurses' sense of professional self‐actualization and what is the role of nursing work environment in prediction of nurses' sense of professional self‐actualization? Is there an association between nurses' sense of professional self‐actualization and their involvement in promotion and marketing of the nursing profession?

Therefore, the study aimed to explore the relationships between nurses' sense of professional self‐actualization, nursing work environment and their involvement in promotion and marketing of the nursing profession.

## 2 PARTICIPANTS, ETHICS AND METHODS

### 2.1 Sample

Inclusion criterion was being a Registered Nurse employed in any healthcare organization (general hospital, community services, psychiatric hospital and geriatric hospital) in the public healthcare sector. Exclusion criteria were being a practical nurse and experience in nursing of less than 2 months. In accordance with Hulley et al. (2013), with power at 0.8, *α* = 0.05 and an anticipated medium effect size, a minimum of 120 subjects were required. Socio‐demographic and occupational characteristics of the sample are shown in Table 1.

**TABLE 1:** Socio‐demographic and occupational characteristics of the sample (*N* = 144)

Variable	*N*	%	*M*	*SD*	Range
Gender					
Male	36	25%			
Female	102	70.8%			
Missing	6	4.2%			
Age			41.4	10.1	24–68
Country of birth					
Israel	64	44.4%			
Former USSR	73	50.7%			
Other	6	4.2%			
Missing	1	0.7%			
Healthcare organization					
General hospital	96	66.7%			
Community services	24	16.7%			
Psychiatric hospital	12	8.3%			
Geriatric hospital	12	8.3%			
Place of employment general hospital					
Medical wards	35	36%			
Intensive care and intensive cardiac care units	21	22%			
Surgical wards	19	20%			
Obstetrics and gynaecology wards	10	10%			
Paediatric ward	9	9%			
Missing	2	2%			
Total	96	100%			
Professional qualification					
RN	37	25.7%			
RN, B.A.	69	47.9%			
RN, M.A.	37	25.7%			
Missing	1	0.7%			
Position					
Staff nurse	103	71.5%			
Clinical team leader	12	8.3%			
Charge nurse	10	6.9%			
Clinical instructor	9	6.3%			
Deputy charge nurse	7	4.9%			
Clinical supervisor	2	1.4%			
Missing	1	0.7%			
Experience in nursing			15.5	10.4	0.2–48
Type of employment					
100%	102	70.8%			
76%–90%	19	13.2%			
51%–75%	20	13.9%			
33%–55%	2	1.4%			
Missing	1	0.7%			

As shown, a convenience sample of 144 nurses working in the public healthcare sector in central Israel participated in the study. Most of them were female (71%, 102), with a mean age of 41.4 [*SD* 10.1, range 24–68]. Approximately half of the sample (51%, 73) were born in the former Soviet Union and the others were Israeli‐born (44%, 63); 26% (37) were Registered Nurses (RNs), 48% (69) were RNs with a bachelor degree (B.A.), and 26% (37) were RNs with a master's degree (M.A.). Most of the participants worked in general hospitals (67%, 96), with medical wards being the most frequent place of employment (36%); 17% (24) worked in the community. The mean participants' seniority in nursing was 15.5 years (*SD* 10.4, range 0.2–48). Most of them worked as staff nurses (72%, 104) and the rest occupied various advanced positions (clinical instructor, clinical team leader, deputy charge nurse, charge nurse, clinical supervisor). Most were employed full‐time (71%, 102).

### 2.2 Instrument

A self‐administered structured questionnaire consisted of four sections:

The first section examined the nurse's s*ocio‐demographic, professional and occupational characteristics* (8 items): gender, age, country of birth, type of healthcare organization, professional qualification, position, experience in nursing and type of employment.

*Nursing work environment* variable was measured using a tool comprised of 20 items from the Revised Nursing Work Index (NWI‐R) (Aiken & Patrician, 2000). These items represent three sub‐scales of the original scale, measuring autonomy, control over the work environment and relationships with physicians. We used the Hebrew version of the NWI‐R (Kagan et al., 2016). The items, for instance, were as follows: “Staff nurses are involved in the internal governance of the hospital,” “There is career development/clinical ladder opportunity,” or “A nurse manager backs up the nursing staff in decision‐making, even if the conflict is with a physician.” Nurses were asked to rank their degree of agreement on a scale from 1 (strongly disagree)–5 (strongly agree). The overall score was represented by the mean. The higher is the overall score, the more the work environment is perceived as supporting and promoting nurses and the nursing profession. Cronbach's alpha for this part of the tool was 0.90.

The third section examined the extent of *nurses' involvement in promotion and marketing of the nursing profession*, targeting the public, nursing community and other healthcare professionals. For this purpose, we used a questionnaire developed in a previous study by Kagan et al. (2015). This part included 17 items (sample items: “Holding discussions with nurses from other fields concerning promotion of the nursing profession,” “Presentation of academic projects to medical and paramedical staff",” “Presentation of the role of a nurse in the field of my work to the public”). Nurses were asked to rank the frequency of their involvement in promotion and marketing of the nursing profession over the last 2 years, on a scale ranging from 1 (never)–6 (weekly). The overall score was represented by the mean. The higher is the score, the more was the nurse involved in promotion and marketing of the nursing profession. Cronbach's alpha was 0.85.

*Professional self‐actualization* variable measurement was based on the Brief Index of Self‐Actualization questionnaire (Sumerlin & Bundrick, 1996). The original tool includes 40 items and refers to seven features of self‐actualizing people. It was developed exclusively from Maslow's composite writings to measure self‐actualization (Sumerlin & Bundrick, 1996). In this study, only the Core Self‐Actualization sub‐section was used. It relates to characteristics that move an individual towards attainment of full potential. They include preparation for the future, contributions to humankind, strength to face the future, happiness, focus, pride in accomplishment and a continued commitment to learning. The questionnaire was adapted for the purposes of this study and included eight items from the original ten (sample items: “I think my present occupation as a nurse contributes to the society", “I enjoy my professional achievements”). Nurses were asked to rank their degree of agreement on a scale ranging from 1 (strongly disagree)–5 (strongly agree). The overall score of this instrument was represented by the mean. The higher is the overall score, the higher is the nurse's sense of professional self‐actualization. Cronbach's alpha was 0.81.

### 2.3 Procedure

The present study is a descriptive cross‐sectional correlational study. The questionnaires were distributed by the researchers, with the aim of reaching nurses employed in as many clinical fields as possible. Nurses were approached in vicinity of their place of employment. The participants were informed about the purpose of the study and assured that their anonymity would be preserved. During March 2017, one hundred sixty questionnaires were distributed. 144 were returned (response rate of 90%).

### 2.4 Statistical analysis

SPSS version 25 (IBM, US) was used to analyse the data. Descriptive statistics were used to present socio‐demographic data distribution and responses to the questionnaire items. As the data were normally distributed, parametric statistics were applied. Pearson correlations and linear regression analyses used to examine the associations between the variables. *t* Tests for independent samples and ANOVA were performed to examine differences between groups. Statistical significance was set at *p* < .05.

### 2.5 Ethical considerations

The study was approved by the Ethics Committee of Tel Aviv University.

### 2.6 Findings

The nurses reported low involvement in activities of promotion and marketing of the nursing profession over the past 2 years (mean = 2.2, *SD* 0.7; 1–6 scale). Of note, all the activities presented in the questionnaire were performed by the nurses with a similar—low—frequency.

As to the nurses' reported sense of professional self‐actualization, it tended to be moderate–high (mean = 4.0, *SD* 0.7). The nursing work environment was ranked with a moderate score (mean = 3.3, *SD* 0.6; on 1–5 scale). In other words, the workplace was viewed as neither supporting nor negative towards the nursing profession.

A statistically significant difference was found in nurses' involvement in promotion and marketing of the nursing profession, according to the professional qualification [*F*_(2,144)_ = 9.0, *p* < .001]. Thus, Registered Nurses with an M.A. degree in nursing reported the highest involvement in marketing activity (mean = 2.6, *SD* 0.7) and then, in descending order, Registered Nurses (mean = 2.2, *SD* 0.7) and Registered Nurses with a B.A. degree in nursing (mean = 2.0, *SD* 0.7). A comparison between nurses in the context of their position revealed significant deterrence. Nurses in managerial positions reported higher involvement (mean = 2.6, *SD* 0.8 vs. mean = 2.1, *SD* 0.7; *t*_(_ *_df_*_=128)_ = 3.7, *p* < .001) and higher sense of self‐actualization (mean = 4.2, *SD* 0.7 vs. mean = 3.9, *SD* 0.7; *t*_(_ *_df_*_=135)_ = 2.1, *p* < .05) than staff nurses.

The correlational analysis was completed in two steps. Firstly, we examined the associations between the socio‐demographic (age) and occupational (experience in nursing) characteristics and the main study variables. The findings reveal that age and experience in nursing were not associated with any of the main study variables. Secondly, the associations between the main study variables were examined. Findings of the correlational analysis are presented in Table 2. Thus, a positive significant correlation between nurses' perception of their work environment and their sense of professional self‐actualization (*r* = .45, *p* < .001) was found. The more nurses perceive their work environment as supportive, the higher is their sense of professional self‐actualization. In addition, a positive correlation between nurses' sense of professional self‐actualization and their involvement in promotion and marketing of the nursing profession (*r* = .32, *p* < .001) was found. The higher is the nurses' sense of professional self‐actualization, the more they are involved in the promotion and marketing activity. Moreover, a positive correlation was found between nurses' perception of their work environment and their involvement in marketing of the nursing profession (*r* = .38, *p* < .001). The more nurses perceive their work environment as supportive, the more they are involved in the promotion and marketing activity.

**TABLE 2:** Pearson correlations between the main study variables (*N* = 144)

Study variables	1	2
1. Perception of the work environment	–	–
2. Sense of professional self‐actualization	0.45*	–
3. Promotion and marketing of the nursing profession	0.38*	0.32*

* *p* < .001.

Variables that were correlated with the main study variables or those that were associated with significant differences in the main study variables were entered into regression. Findings of a regression analysis revealed that nurses' perception of their work environment and holding an M.A. degree in nursing explained some of the variance in their involvement in promotion and marketing of the nursing profession. In contrast, nurses' sense of professional self‐actualization and occupying managerial positions did not contribute to the explanation of this variance (Table 3). The model explained 27% of the variance in nurses' involvement in promotion and marketing of the nursing profession [*R* ^2^ = 0.27, *F*_(4,113)_ = 10.4, *p* < .001]. Performing the regression in the stepwise method revealed that nurses' perception of their work environment alone explained 14% of the variance in their involvement in the marketing activity [*R* ^2^ = 0.14, *F*_(1,116)_ = 18.60, *p* < .001]. The addition of holding an M.A. degree in nursing to regression explained additional 10% in the variance in nurses' involvement in the marketing activity [*R* ^2^ = 0.24, *F*_(2,115)_ = 17.7, *p* < .001]. Findings of an additional regression analysis revealed that nurses' perception of their work environment explained some of the variance in their sense of professional self‐actualization (*t* = 5.27, *B *= 0.48, *SE *= 0.09, Beta = 0.43, *p *< .001), while occupying an advanced position did not. The model explained 21% of the variance in nurses' sense of professional self‐actualization [*R* ^2^ = 0.21, *F*_(2,121)_ = 16.5, *p* < .001].

**TABLE 3:** Results of a regression analysis for exploring the factors associated with nurses' involvement in promotion and marketing of the nursing profession (*N* = 144)

Variable	*B*	*SE*	*β*	*t*	*p*‐value
Perception of the work environment	0.29	0.10	0.26	2.85	.005
Sense of professional self‐actualization	0.12	0.09	0.12	1.35	.18
Holding an M.A. degree in nursing	0.41	0.14	0.26	3.01	.003
Occupying advanced managerial/clinical position	0.24	0.14	0.15	1.70	.09

## 3 DISCUSSION

This study explored associations between nurses' sense of professional self‐actualization, work environment and their involvement in promotion and marketing of the nursing profession. In the present study, nurses reported minimal involvement in activity of promotion and marketing of the nursing profession, in consistence with a previous study (Kagan et al., 2015). The study findings suggest that nurses' sense of professional self‐actualization does not possibly play a significant direct role in their involvement in this activity. This is despite a certain evidence in previous research of the association of professional self‐actualization with several important concepts, such as nurses' job satisfaction (Banks & Bailey, 2010; Gilles et al., 2014; McGlynn et al., 2012), which has been shown to be associated with nurses' involvement in promotion and marketing of the nursing profession (Kagan et al., 2015). In other words, a nurse, who feels that he/she has attained her potential in nursing, will not necessarily be willing to be involved in promotion or marketing of the nursing profession and probably, other conditions need to be fulfilled, for a nurse to be involved in this activity. It should be noted that our findings do not exclude the possibility that the association between nurses' sense of professional self‐actualization and their involvement in promotion or marketing of the nursing profession may be moderated by certain factors, but this has not been explored in the present study, warranting further research.

In contrast, the study findings emphasize the significant role of work environment in the explanation of nurses' involvement in promotion and marketing of the nursing profession. In the present study, nurses who perceived their work environment as more supporting and promoting the nursing profession were more involved in promotion and marketing of the nursing profession. This finding supports the hypothesis raised in a previous study concerning a possible association between nurses' work environment and their involvement in the promotion and marketing activity (Kagan et al., 2015). However, the present study suggests that aside from the organizational climate, that stresses the significance of professional image and public relations, it is important that the nursing work environment be positive, comfortable and supportive. It seems that such environment may motivate nurses to promote their profession.

In the present study, Registered Nurses with an M.A. degree in nursing reported higher involvement in promotion and marketing of the nursing profession, than Registered Nurses and Registered Nurses with a B.A. degree in nursing. By completion of their academic studies for an advanced degree, Registered Nurses with an M.A. degree may be more aware of the need to promote and market their profession, as well as have more tools to do it.

In addition, nurses in advanced managerial/clinical position reported higher involvement in the promotion and marketing activity as compared with staff nurses. At first glance, nurses occupying any administrative or supervising position may be at a more comfortable position to take an active role in promotion and marketing of the nursing profession, compared with staff nurses. However, as revealed by the regression analysis, only holding the M.A. degree in nursing explained some of the variance in nurses' involvement in the promotion and marketing activity, while occupying an advanced managerial or clinical position did not. These findings suggest that awareness and appropriate tools, rather than the position per se, are the factors that are associated with nurses' involvement in the promotion and marketing activity. Of note, the effect of holding an M.A. degree in nursing on nurses' involvement in this activity was like the effect of the work environment.

In addition, this study explored antecedents of nurses' sense of professional self‐actualization. In the present study, nurses who perceived their work environment as more supportive reported higher sense of professional self‐actualization. This finding contributes to the expansion of knowledge on the role of nurses' work environment in explanation of their sense of professional self‐actualization, as has been previously suggested in the nursing literature (Casida & Parker, 2011; Paris & Terhaar, 2010). The present study confirms that when a nurse perceives working conditions as comfortable, experiences safety, belonging and esteem, while having a nursing manager who promotes nurses and the nursing profession, he/she will more likely perceive having attained his/her potential in nursing. It should be noted that in the present study, the nurses' reported sense of professional self‐actualization tended to be moderate–high. In this study, nurses tended to perceive the work environment as not harming the nursing profession (though as not necessarily supportive) and this finding may explain, at least in part, the relatively high sense of professional self‐actualization reported by nurses in the present study.

In the present study, nurses occupying any administrative or supervising position reported higher sense of professional self‐actualization than staff nurses, suggesting that career advancement may be associated with a greater feeling of having attained his/her potential in nursing. However, this difference was small and its effect on the sense of professional self‐actualization was found insignificant, as revealed by the regression analysis. This suggests that the feeling of having attained his/her potential in nursing may be present independently of career advancement and possibly depends on how an individual nurse defines what is his/her potential in nursing.

In addition, in the present study, no difference was found in nurses' sense of professional self‐actualization, across the three different professional qualifications (Registered Nurses and Registered Nurses with a B.A. or an M.A. degree in nursing). This suggests that a nurse's feeling of having attained his/her potential in nursing may be present independently of professional qualification and again, and supports the assumption that the feeling of having attained his/her potential in nursing may depend on how an individual nurse defines what is his/her potential in nursing.

However, lack of association between occupying an advanced managerial or clinical position, professional qualification and a sense of professional self‐actualization in nursing, raises a concern as to whether these findings indicate a certain tendency to stagnation among nurses, being satisfied with what they have and where they are and maybe, certain unwillingness to move forward. The following question is whether, in this condition, nurses will have the willingness to promote and market their profession. This possibly may clarify why nurses' professional self‐actualization did not contribute to the explanation of the variance in their involvement in promotion and marketing of the nursing profession.

Finally, in the present study, nurses' age and experience in nursing were not related to their sense of professional self‐actualization. In other words, both younger and older nurses, as well as nurses of different durations of experience in nursing, reported similar level of professional self‐actualization. This finding conflicts with the findings of a previous study where older individuals reported higher self‐actualization and somewhat contradicts Maslow's Hierarchy of Needs Theory, which suggests that as people mature and develop, self‐actualization becomes their most prominent need (Ivtzan et al., 2013). This finding may also possibly indicate a certain tendency to stagnation among nurses.

### 3.1 Study limitations

The present study has several limitations. The convenience sampling method limits the generalizability of the study results to the general population of nurses. In addition, the study design does not permit to establish cause‐and‐effect. Therefore, the findings should be interpreted with caution. Finally, due to a small number of respondents in the different clinical fields, it was impossible to compare involvement in the promotion and marketing activity, nor sense of professional self‐actualization, between the clinical fields, thus warranting further research.

## 4 CONCLUSIONS AND IMPLICATIONS

The present study suggests that nurses' professional self‐actualization does not play a significant direct role in their involvement in promotion and marketing of the nursing profession, possibly, due to a certain tendency to stagnation among nurses. However, this study identified several other antecedents of nurses' involvement in the promotion and marketing of the nursing profession, which are work environment, as well as nurses' awareness and having the appropriate tools for performing the promotion and marketing activity. These findings implicate, that to foster nurses' involvement in the promotion and marketing activity, a work environment that supports and promotes nurses and the nursing profession should be established and steps should be taken to promote nurses' awareness and help them acquire the appropriate tools, for example by encouraging nurses to acquire an advanced degree in nursing. In addition, the work environment has been identified as a significant antecedent of nurses' professional self‐actualization, suggesting that establishing a work environment that supports and promotes nurses and the nursing profession will increase nurses' feeling of having attained their potential in nursing. Further research is needed to explore how nurses define their potential in nursing and whether indeed there is a certain tendency to stagnation.

## CONFLICT OF INTEREST

The authors declare that there is no conflict of interest.

## AUTHOR CONTRIBUTIONS

YH, YG and IK: Study design.YG: Data collection.YH: Data analysis.IK: Study supervision.YH, YG and IK: Manuscript writing.IK and RWM: Critical revisions for important intellectual content. We confirm that all authors have made substantial contributions to all of the following: (1) the conception and design of the study, or data gathering, or data analysis and interpretation, (2) drafting the article or revising it critically for important intellectual content, and (3) final approval of the version to be submitted.

## Data Availability

The data that support the findings of this study are available from the corresponding author, [IK], upon reasonable request.
